# A novel acidic polysaccharide from blackened jujube: Structural features and antitumor activity *in vitro*

**DOI:** 10.3389/fnut.2022.1001334

**Published:** 2022-09-15

**Authors:** Guifeng Zhang, Chuang Liu, Rentang Zhang

**Affiliations:** Key Laboratory of Food Processing Technology and Quality Control of Shandong Higher Education Institutes, College of Food Science and Engineering, Shandong Agricultural University, Tai'an, Shandong, China

**Keywords:** blackened jujube, polysaccharides, structural features, antitumor activity, mechanism

## Abstract

Liver cancer is one of the most common cancers, with increasing trends in incidence and mortality. A novel acidic polysaccharide (BJP-2) obtained from blackened jujube was extracted by hot water followed by chromatographic purification employing DEAE-cellulose 52 and Sephadex G-100 column. And then BJP-2 was identified by SEC-MALLS-RI, GC-MS, methylation and NMR for the following characteristics: molecular weight of 6.42 × 10^4^ Da, monosaccharide composition of glucuronic acid (GalA), arabinose (Ara), galactose (Gal), rhamnose (Rha), xylose (Xyl), glucuronic acid (GlcA), glucose (Glc), fucose (Fuc) and mannose (Man) with the percentage of 39.78, 31.93, 16.86, 6.43, 1.86, 1.28, 1.02, 0.61, and 0.23%, as well as the main chain of → 5)-α-L-Ara*f* (1 → 4)-β-D-Gal(1 → , T-α-L-Ara*f* (1 → 4)-β-D-Gal(1 → , and → 4)-α-L-6MeGalA*p*(1 → . The effect of BJP-2 on the apoptosis of HepG2 cells and its anti-tumor mechanism were further explored. The analysis by MTT and flow cytometry showed that BJP-2 suppressed cell proliferation by inducing apoptosis in a concentration-dependent manner. Cell scratching and Transwell revealed that BJP-2 was able to block the invasion and metastasis of tumor cells. Western blot results demonstrated that BJP-2 exhibited antitumor activity through a mitochondria-dependent pathway, as evidenced by overexpression of Bax, Cleaved Caspase-3/Caspase-3 and Cleaved Caspase-9/Caspase-9 and downregulation of Bcl-2. Therefore, BJP-2 has broad research prospects as a tumor preventive or therapeutic agent.

## Introduction

At present, malignant tumors are one of the most serious diseases that threaten human health and life, and are classified into liver cancer, lung cancer, breast cancer, colorectal cancer and so on, according to their sites of development (1–3). Of these, liver cancer has a high incidence in China, accounting for more than 50% of new liver cancer patients worldwide each year, and its danger should not be underestimated (4). With the advancement of medical technology, surgery, radiotherapy and chemotherapy are three key and effective means of cancer treatment (5). However, many of the side effects can lead to a decrease in the immune function of the patient's organism, while the drug relieves symptoms and extends the patient's life (6). Therefore, the investigation and discovery of novel antitumor drugs with low cost, low toxicity and high efficiency are of vital importance.

Plant polysaccharides, a polymeric sugar polymer carbohydrate consisting of more than 10 monosaccharides extracted from plants, has various pharmacological effects such as antitumor, anti-inflammatory, antioxidant, immunomodulatory and hypoglycemic (7–9). Among them, the antitumor activity of plant polysaccharides has been widely studied and recognized, and the mechanisms include prevention of tumorigenesis, activation of immune response, direct inhibition and killing of tumor cells, enhancement of the body's anti-free radical effect and inhibition of angiogenesis in tumor tissues (10).

Jujube (*Ziziphus jujuba* Mill.) contains abundant active ingredients such as polysaccharides, flavonoids, polyphenols and saponins, and thus has versatile health and medicinal values (11, 12). The cell experiments showed that a polysaccharide fraction (HJP3) obtained from *Zizyphus jujuba cv. Muzao* significantly suppressed the proliferation of HepG2 cells, but was not cytotoxic to non-tumor cell lines, and the analysis might be that HJP3 exerted anti-tumor activity directly and induced apoptosis of tumor cells (13). *Zizyphus jujuba cv.Ruoqiangzao* seed polysaccharides, obtained by ultrasound-assisted-hot water extraction, exhibited concentration-dependent inhibition of proliferation of HeLa cells, probably through induction of apoptosis (14). A *Zizyphus jujuba cv. Goutouzao* polysaccharide with antioxidant activity that prevented LoVo cells growth, which was mediated by inducing apoptosis and enhancing intracellular reactive oxygen species secretion (15). Blackened jujube, a new kind of jujube processing product, is made by fermenting dried jujube in a high temperature and high humidity environment (16–18). Blackened jujube polysaccharides purified from *Z. jujuba cv. Hamidazao* have previously been reported to exert excellent antioxidant capacity *in vitro* compared to dried jujube, including free radical scavenging and total reducing capacity (19). *Z. jujuba cv. Huizao* polysaccharides have been shown to have immunomodulatory effects by improving serum hemolysin formation and increasing the phagocytic capacity of macrophages (20). Taking into account the close relationship between antitumor ability and immunomodulatory activity, we speculated that black jujube polysaccharide had good anti-tumor activity. To the best of our knowledge, the anti-tumor activity of polysaccharides from blackened jujube has never been investigated. This greatly limited their application in the field of pharmaceuticals and functional foods. Therefore, it is quite necessary to evaluate the structural characteristics and anti-tumor activity of polysaccharides from blackened jujube (made from *Z. jujuba cv. Huizao*).

The highest mass yield of BJP-2 (25.8%) was obtained by using the earlier described technique which employed hot water extraction followed by a DEAE-cellulose 52 and Sephadex G-100 column (21). In this study, the structure of BJP-2 was systematically presented by multi-angle laser light scattering combined with SEC and differential refractive index detector (SEC-MALLS-RI), gas chromatography-mass spectrometry (GC-MS), methylation, and nuclear magnetic resonance (NMR, 1D and 2D). Furthermore, human hepatocellular carcinoma cells (HepG2) were used as a model to investigate its anti-tumor ability and mechanism.

## Materials and methods

### Materials

*Z. jujuba cv. Huizao* fruits were provided by Guorentang Food Technology Co., Ltd. (Shandong, China). HepG2 cells were obtained from Shandong Analysis and Testing Center (Jinan, China). High glucose Dulbecco's modified eagle medium (DMEM), fetal bovine serum (FBS), penicillin and streptomycin were provided from Gibco Biotechnology Co., Ltd. (Grand Island, New York, USA). Monosaccharide standards (Fuc, Rha, Ara, Gal, Glc, Xyl, Man, Fru, Rib, GalA, GulA, GlcA and ManA), 3-(4,5-dimethylthiazol-2-yl)-2,5-diphenyltetrazolium bromide (MTT), trifluoroacetic acid (TFA), Mitomycin C and NaBH_4_ were bought from Sigma-Aldrich (St. Louis, MO, USA).Bicinchoninic Acid (BCA) protein kit and protein extraction kit were purchased from Beyotime Biotechnology Co., Ltd. (Shanghai, China). The Annexin V-Fluorescein Isothiocyanate/Propidium Iodide (FITC/PI) Apoptotic Cell Detection Kit and antibodies (Bcl-2, Bax, Caspase-3, Cleaved Caspase-3, Caspase-9 and Cleaved Caspase-9) were obtained from Wanlei Biotechnology Co., Ltd. (Shenyang, China). All other chemicals and reagents were of analytical reagent grade.

### Structural features of BJP-2

#### Chemical analysis

The chemical composition of BJP-2 was determined based on the previous method (22, 23). To determine total sugar content, the phenol-sulfuric acid method was used, with glucose as the standard. Using bovine serum albumin as the reference, the Bradford method was adopted to assay the protein content. For the determination of total phenol content, the Folin-Ciocalteu colorimetric method was applied. The total flavonoid content was estimated with sodium nitrite-aluminum nitrite method, using rutin as the standard.

#### Molecular weight distribution

As described by previous studies (24), briefly, the solution of BJP-2 (1 mg/ml) was configured with 1 M NaNO_3_ as a solvent and passed through a filter (0.45 μm). A DAWN HELEOS-II laser photometer (Wyatt Technology Co., Santa Barbara, CA, USA) equipped with three tandem columns (300 × 8 mm, Shodex OH-pak SB-805, 804 and 803; Showa Denko K.K., Tokyo, Japan) was used for the determination under the following conditions: column temperature of 45°C, flow rate of 0.4 ml/min, injection volume of 100 μl, and mobile phase A of 0.1 M NaNO_3_. Data were acquired and processed using ASTRA6.1 (Wyatt Technologies Inc., USA).

#### Monosaccharide composition

5.00 mg of BJP-2 powder was transferred to a chromatographic sample bottle and then hydrolyzed by TFA (2 M, 1 ml) for 2 h at 121°C, after which it was repeatedly washed three times with methanol and blow-dried with nitrogen. The residue was re-dissolved in deionized water and purified through a 0.22 μm microporous filter before determination on an HPAEC-PAD (Thermo Fisher ICS-5000+, USA) equipped with a Dionex™ CarboPac™ PA-20 chromatography column (Dionex, 3 × 150 mm). Conditions: flow rate, 0.5 ml/min; injection volume, 5 μl; solvent system, B: (0.1 M NaOH, 0.2 M NaAc); gradient program, 95:5 V/V at 0 min, 80:20 V/V at 30 min, 60:40 V/V at 30.1 min, 60:40 V/V at 45 min, 95:5 V/V at 45.1 min, 95:5 V/V at 60 min (25). The different monosaccharides were identified and quantified based on the retention time and peak area of the monosaccharide standards.

#### Methylation analysis

As previously reported data (19), the BJP-2 solution (10 mg/ml, 1 ml) was added to carbodiimide (100 mg/ml, 1 ml) for 2 h and then reacted with imidazole and NaBD_4_ (10 mg/ml, 1 ml) for 3 h, respectively. The mixture was terminated by acetic acid (10 μl), followed by dialysis and freeze-drying. Next, the sample was dissolved by DMSO (500 μl) and then treated by methylation with NaOH (50 μl) and methyl iodide solutions for 30 min and 1 h, respectively. The target, obtained by dichloromethane extraction followed by nitrogen flow drying, was hydrolyzed by TFA (2 M, 100 μl) at 121°C for 1.5 h and then treated by ammonia (2 M, 50 μl) and NaBD_4_ (1 M, 50 μl) at room temperature for 2.5 h. After termination by acetic acid (20 μl)and blowing dry under nitrogen, the resulting sample was acetylated for 2.5 h at 100°C using acetic anhydride (250 μl) and finally extracted with dichloromethane (500 μl). A gas chromatograph-mass spectrometer (GC-MS, Agilent 7890A-5977B, Agilent, Santa Clara, CA, USA) equipped with a BPX70 GC column (30 cm × 0.25 mm × 0.25 μm) was employed. GC parameters: injection volume of 1 μl, splitting ratio of 10:1, carrier gas of high-purity helium, initial temperature of 140°C held for 2.0 min, program of 3°C/min, 230°C held for 3 min. MS parameters: a mode of full scan and a mass scan range setting of 30–600 m/z.

#### NMR spectroscopy analysis

1D NMR spectra (^1^H and ^13^C) and 2D NMR spectra [correlation spectroscopy (COSY), heteronuclear single quantum coherence (HSQC), heteronuclear multiple bond coherence (HMBC) and nuclear overhauser effect spectroscopy (NOESY)] were performed by high-resolution AVANCE III 600 NMR spectrometer (Bruker, Germany). After dissolving 30 mg of BJP-2 in D_2_O, it was added to the NMR tube, and then the spectra were recorded at 25°C.

### Antitumor activity of BJP-2 *in vitro*

#### Cell culture and viability

DMEM medium containing penicillin (100 U/ml) with 10% FBS, 1% penicillin and 1% streptomycin was used to incubate HepG2 cells at 37°C with 5% CO_2_. The effects of BJP-2 on the viability of HepG2 cells were assayed by MTT assay (26). HepG2 cells (5 × 10^3^ cells/well) were stimulated for 48 h with different concentrations of BJP-2 (0, 50, 100, 200, 400, 800 μg/ml) after cells were cultured overnight in 96-well plates. Reaching time, the medium was removed and 20 μl MTT was added to each well, and then placed in an incubator at 37°C and 5% CO_2_ for 4 h. After that, the cell supernatant was aspirated and 150 μl of Formanzan was added, and the optical density (OD) of the reaction solution at 570 nm was recorded on an microplate reader (ELX-800, Biotek, USA). Cell viability was computed as follows:


(1) $$Cell\;viability\;=\;\frac{ODsample}{ODblank}\;\times \;100\%$$

#### Apoptosis detection by flow cytometry

Cells were cultured in 6-well plates at a quantity of 5 × 10^5^, referring to Section Cell culture and viability. After centrifugation (150 g, 5 min) and aspiration of the supernatant, apoptosis was examined using Annexin V-FITC/PI Apoptosis Detection Kit based on the instructions. The flow cytometer (NovoCyte, ACEA, USA) was utilized for the following analysis.

#### Scratch assay

Cells were scratched using a sterile pipette (200 μl), and then the cell surface was washed once with serum-free medium and observed and photographed under a microscope (100 × , IX53, Olympus, Japan). Replaced with serum-free medium, cells were kept in an incubator for 24 h under 37°C and 5% CO_2_ and then photographed and recorded. Mitomycin C (1 mg/ml) was employed to exclude the interference caused by proliferation on migration (27). The cell migration rate was calculated according to the following equation:


(2) $$Cell\;migration\;rate\;=\;1\;-\;\frac{scratch\;width\;of\;48\;h}{scratch\;width\;of\;0\;h}\;\times \;100\%$$

#### Transwell assay

The Transwell chambers (LABSELECT, Anhui, China) were placed in 24-well plates and coated with Matrigel gel (Corning, New York, USA) after overnight thawing at 4°C, and then incubated for 2 h at 37°C (28). Cell cultures from each group were discarded, washing twice with PBS, after which cells were digested by adding trypsin. Resuspension of cells in serum-free medium and adjustment of cell numbers. The Transwell inserts containing Matrigel gel were placed in a 24-well plate, with 800 μl of culture medium containing 10% FBS in the lower chamber and 200 μl of cell suspension in the upper chamber, respectively, and adjusted the cell concentration to 6 × 10^4^ cells/well and placed in the incubator at 37°C. Transwell inserts were washed twice with PBS and fixed in 4% paraformaldehyde (Aladdin, Shanghai, China) at room temperature for 20 min. After that, they were stained with 0.5% crystalline violet (Amresco, USA) staining solution for 5 min, rinsed with distilled water, and observed under an inverted microscope (200 ×).

#### Western blot

Western blot analysis based on the method of literature (29). Briefly, proteins were isolated and quantified using protein extraction and BCA protein assay kits, respectively. Various concentrations of polyacrylamide gels (5–14%) were prepared and subjected to protein electrophoresis, after which the proteins were transferred to polyvinylidene difluoride (PVDF) film. PVDF films were removed and immersed in Tris-buffered saline with Tween 20 (TBST) with shaking for 5 min, then the membrane were immersed in 5% (m/v) skim milk powder solution with shaking for 1 h. Conditions of the primary antibody incubation were Bcl-2 (1:500), Bax (1:1,000), Caspase-3/Cleaved Caspase-3 (1:500), Caspase-9/Cleaved Caspase-9 (1:1,000) and β-actin (1:400), overnight at 4°C. After that, the films were removed from the hybridization bag and soaked in TBST for four washes, and then the goat anti-rabbit IgG HRP secondary antibodies were incubated at a dilution ratio of 1:5,000 for 45 min at 37°C. After six times washing, the membranes were added ECL reagent, and were photographed by WD-9413B imaging system (Beijing LIUYI Biotechnology Co., Ltd., Beijing, China).

### Statistical analysis

Results are denoted as mean ± standard deviation (SD). The significance of differences was evaluated using the one-way analysis of variance (ANOVA) followed by Tukey's test by SPSS software at *p* < 0.05.

## Results and discussion

### Basic chemical composition and molecular weight

**Figure 1:**
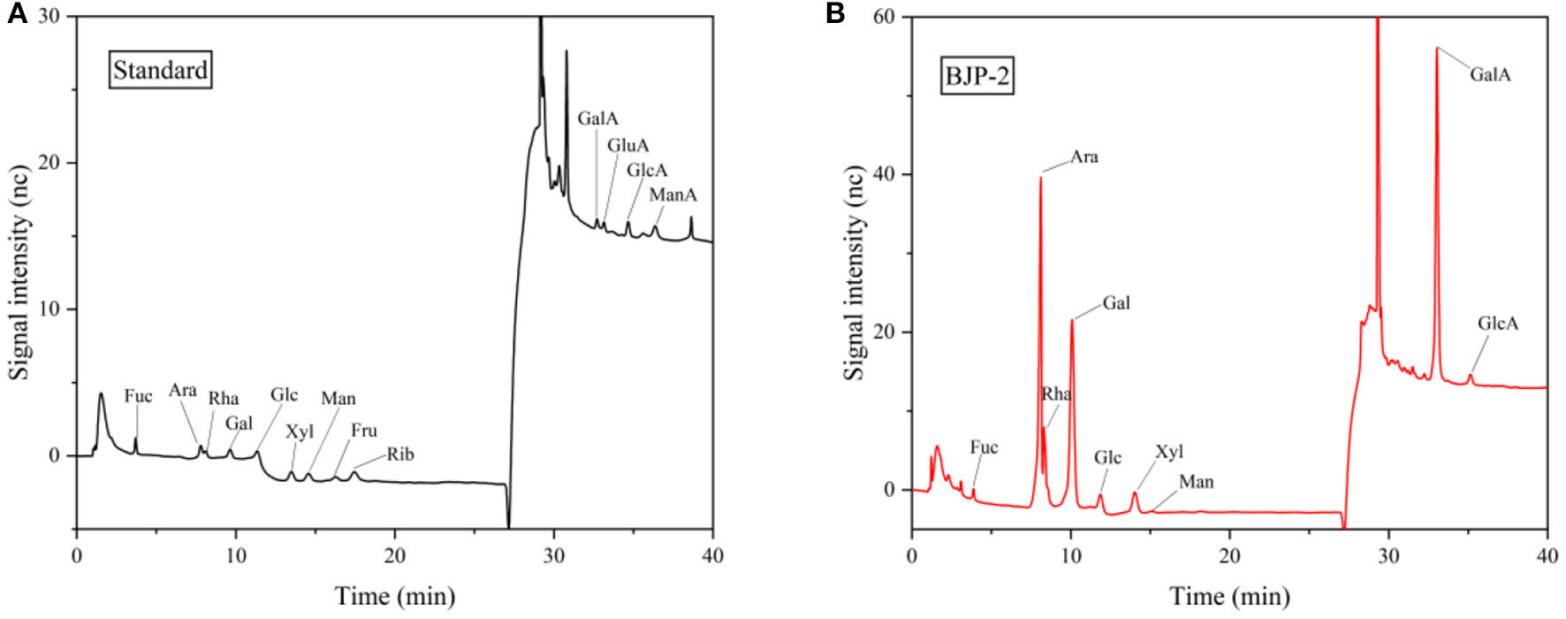
GC-MS analysis of the standard monosaccharides **(A)** and BJP-2 **(B)**.

The total sugar and total flavonoids of BJP-2 were 92 ± 0.05% and 0.81 ± 0.01%, respectively. Its total phenols and protein content were 1.68 ± 0.01% and 0.52 ± 0.01%. The average molecular weight of BJP-2 was 6.42 × 10^4^ Da and the polydispersity index was 3.797. The BJP-2 was principally comprised of GalA (39.78%), Ara (31.93%), Gal (16.86%) and Rha (6.43%), and contained a small amount of Xyl (1.86%), GlcA (1.28%), Glc (1.02%), Fuc (0.61%) and Man (0.23%), showing that BJP-2 was a acidic polysaccharide (Figures 1A,B). Compared to the *Z. jujuba cv. Huizao* polysaccharide (HP-2), they both consisted mainly of GalA, Ara, and Gal, but possessed different percentages, and the molecular weight of BJP-2 (64.2 kDa) was smaller than that of HP-2 (111 kDa) (20). This phenomenon was explained as being caused by blackened jujube during processing, but the specific changes still depend on subsequent systematic research.

### Methylation analysis of BJP-2

The linkage types and ratios of glycosidic bonds are usually determined by methylation analysis. As summarized in Table 1, the BJP-2 were identified as containing seventeen derivatives. Among them, (1 → 4)-linked-Gal*p*A was the most dominant linkage pattern (46.11%), followed by Ara*f* (1 → (12.94%), → 4) Gal*p* (1 → (11.60%) and → 5) Ara*f* (1 → (8.31%), together accounting for 78.96% of the total methylated sugars. Meanwhile, some of the discrepancies between the above results and the analysis of monosaccharide composition were explained by the fact that the dialysis step of acidic sugars when undergoing pre-treatment could have an effect on the results. However, the qualitative and quantitative analyses of the monosaccharide composition of BJP-2 were reliable, and the qualitative results of the methylation results on the type of glycosidic bonds and the mode of attachment were also correct (30). The structure of BJP-2 was further confirmed by NMR spectra.

**Table 1:** Results of methylation analysis of BJP-2.

**Retention time (min)**	**Linkage types**	**Methylated sugars**	**Molar ratio (%)**
5.459	t-Rha*p*	1,5-di-O-acetyl-6-deoxy-2,3,4-tri-O-methyl rhamnitol	0.725
5.72	t-Ara*f*	1,4-di-O-acetyl-2,3,5-tri-O-methyl arabinitol	12.943
6.988	t-Ara*p*	1,5-di-O-acetyl-2,3,4-tri-O-methyl arabinitol	1.010
8.452	2-Rha*p*	1,2,5-tri-O-acetyl-6-deoxy-3,4-di-O-methyl rhamnitol	1.565
9.575	t-Gal*p*	1,5-di-O-acetyl-2,3,4,6-tetra-O-methyl galactitol	3.539
9.575	t-Gal*p*A	1,5-di-O-acetyl-2,3,4,6-tetra-O-methyl galactitol	3.517
10.214	5-Ara*f*	1,4,5-tri-O-acetyl-2,3-di-O-methyl arabinitol	8.306
12.016	2,4-Rha*p*	1,2,4,5-tetra-O-acetyl-6-deoxy-3-O-methyl rhamnitol	3.900
12.46	3-Gal*p*	1,3,5-tri-O-acetyl-2,4,6-tri-O-methyl galactitol	0.959
13.365	4-Gal*p*	1,4,5-tri-O-acetyl-2,3,6-tri-O-methyl galactitol	11.592
13.365	4-Gal*p*A	1,4,5-tri-O-acetyl-2,3,6-tri-O-methyl galactitol	46.105
13.666	4-Glc*p*	1,4,5-tri-O-acetyl-2,3,6-tri-O-methyl glucitol	0.448
13.666	4-Glc*p*A	1,4,5-tri-O-acetyl-2,3,6-tri-O-methyl glucitol	0.297
15.055	6-Gal*p*	1,5,6-tri-O-acetyl-2,3,4-tri-O-methyl galactitol	1.199
15.328	3,4-Gal*p*	1,3,4,5-tetra-O-acetyl-2,6-di-O-methyl galactitol	0.894
15.328	3,4-Gal*p*A	1,3,4,5-tetra-O-acetyl-2,6-di-O-methyl galactitol	0.889
18.472	3,6-Gal*p*	1,3,5,6-tetra-O-acetyl-2,4-di-O-methyl galactitol	2.112

### NMR spectral analysis of BJP-2

**Figure 2:**
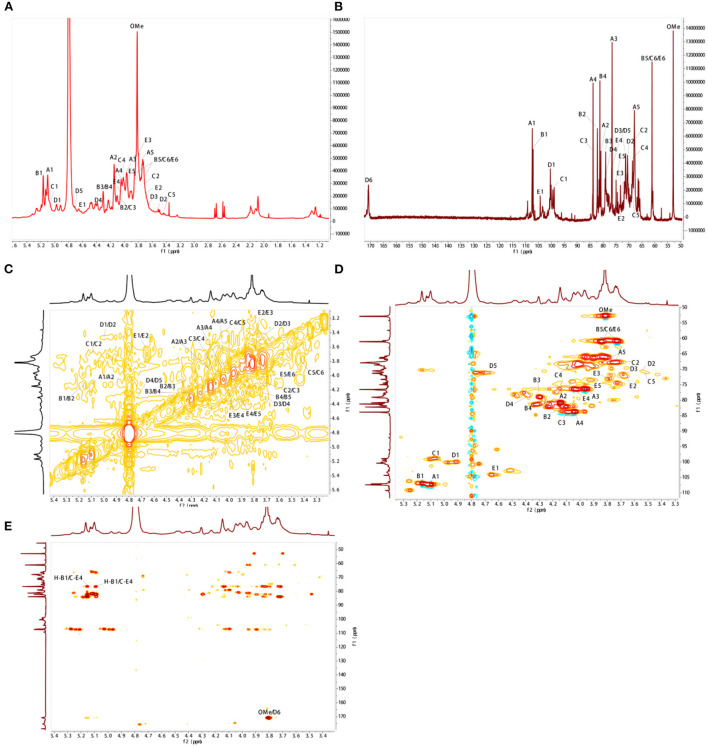
NMR spectral analysis of BJP-2. **(A)** ^1^H-NMR; **(B)** ^13^C-NMR; **(C)** ^1^H-^1^H COSY; **(D)** HSQC; **(E)** HMBC.

As shown in Figure 2A, BJP-2 exhibited five major anomeric proton signals (δ 5.10, 5.16, 5.07, 4.97, and 4.65) in the ^1^H NMR spectrum, which were labeled as A, B, C, D, and E, respectively. Meanwhile, the chemical shifts of the protons from C-2 to C-6 of the residues were mainly distributed in the range of δ 4.75–3.40 ppm. Referring to the HSQC spectrum, there were five heterocephalic signals at δ 107.48, 107.05, 98.92, 100.39, and 104.33 ppm at the ^13^C NMR spectrum (Figure 2B). Based on the reported literature, these ^1^H and ^13^C signals were assigned to the ^1^H-^1^H COSY (Figure 2C) and HSQC (Figure 2D) spectra (31–34). Table 2 provided information on the signal assignments of the protons and carbons for the five major residues in BJP-2. The anomeric proton and carbon of residue A produced the chemical shifts at δ_C_ 107.48/δ_H_ 5.10 ppm (C-1), δ_C_ 80.88/δ_H_ 4.14 ppm (C-2), δ_C_ 76.53/δ_H_ 3.95 ppm (C-3), δ_C_ 83.82/δ_H_ 4.04 ppm (C-4) and δ_C_ 67.91/δ_H_ (3.75/4.02 ppm) (C-5), respectively. Summarizing these NMR data, it was inferred that it belongs to → 5)-α-L-Ara*f* (1 → (31). Furthermore, B–E were designated as T-α-L-Ara*f* (1 → , T-β-D-Gal*p*(1 → , → 4)-α-L-GalA*p*(1 → , → 4)-β-D-Gal (1 → (32, 33). Additionally, signals of OMe were also observed as δ_H_ 3.81/δ_C_ 52.91. Taking the results of methylation analysis into consideration, it was supposed that the presence of terminal signal suggested as T-α-L-Ara*f* (1 → and T-β-D-Gal*p*(1 → in BJP-2 (19). In the HMBC spectrum, some inter-residual cross peaks were found: A H-1 to E C-4, B H-1 to E C-4; OMe to δ_C_ 170.81 (Figure 2E). In summary, the main chains of BJP-2 were → 5)-α-L-Ara*f* (1 → 4)-β-D-Gal(1 → , T-α-L-Ara*f* (1 → 4)-β-D-Gal(1 → , and → 4)-α-L-6MeGalA*p*(1 → , with two different terminal residues of T-α-L-Ara*f* (1 → and T-β-D-Gal*p*(1 → .

**Table 2:** Assignments of ^1^H and ^13^C NMR spectra for BJP-2.

			**1**	**2**	**3**	**4**	**5**	**6**
A	→ 5)-α-L-Ara*f*(1 →	C	107.48	80.88	76.53	83.82	67.91	
		H	5.10	4.14	3.95	4.04	3.75/4.02	
B	T-α-L-Ara*f*(1 →	C	107.05	82.20	79.08	81.23	61.05	
		H	5.16	4.09	4.28	4.30	3.84/3.75	
C	T-β-D-Gal*p*(1 →	C	98.92	67.73	83.87	68.61	70.69	61.05
		H	5.07	3.70	4.09	3.99	3.53	3.84/3.75
D	→ 4)-α-L-Gal*p*A(1 →	C	100.39	70.55	71.32	79.00	71.24	170.81
		H	4.97	3.50	3.68	4.38	4.66	
E	→ 4)-β-D-Gal (1 →	C	104.33	71.78	73.27	76.69	74.98	61.05
		H	4.65	3.68	3.78	4.05	3.94	3.84/3.75
OMe	52.91/3.81							

### Antitumor effects *in vitro*

#### Cell viability of BJP-2

**Figure 3:**
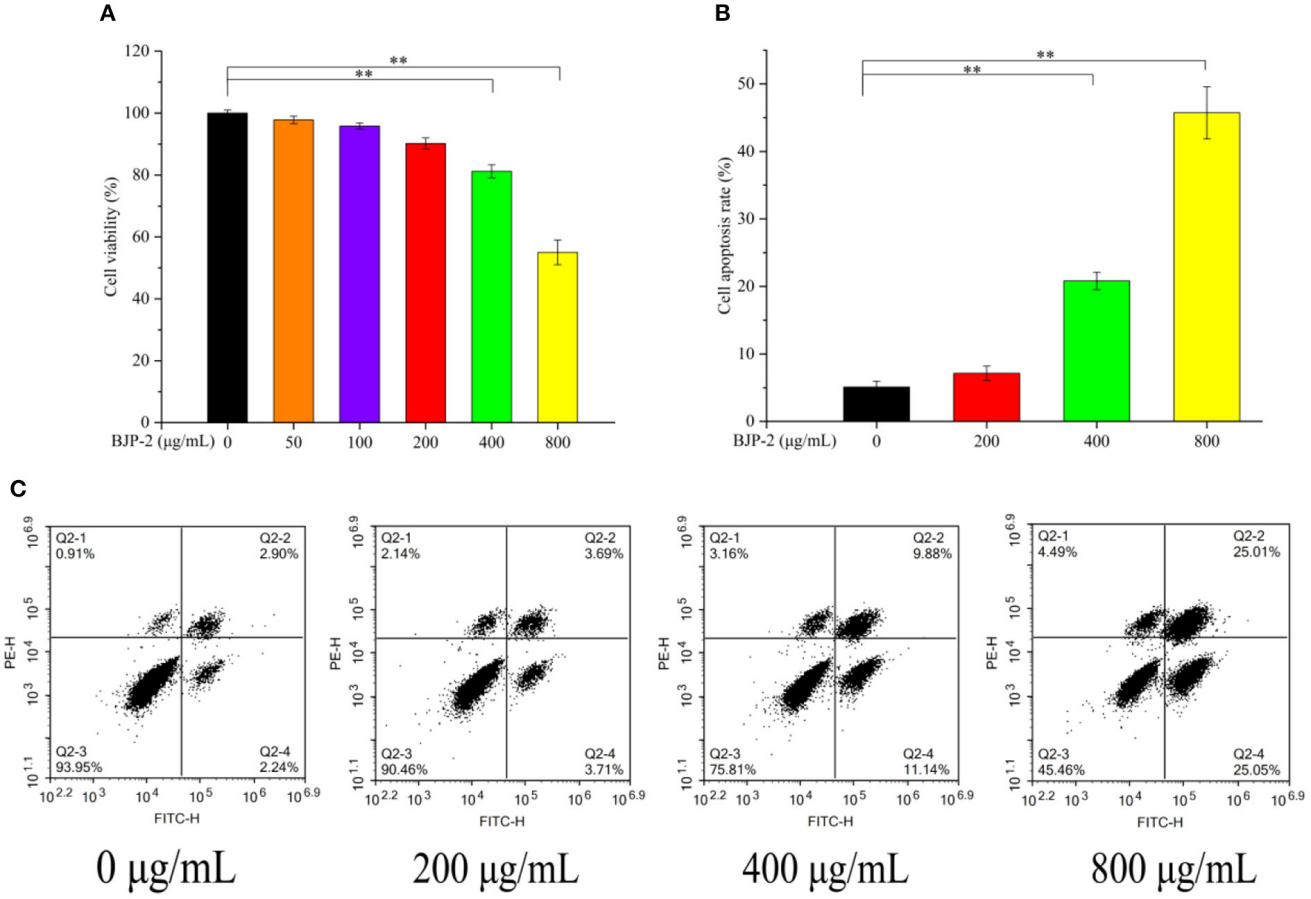
**(A)** Changes of HepG2 cell viability after 48 h incubation with different concentrations of BJP-2. **(B)** Column bar graph of apoptotic HepG2 cells. **(C)** The apoptosis of HepG2 cells treated with BJP-2 at different concentrations for 48 h and were detected by flow cytometry. Values are expressed as mean ± SD. *p < 0.05; **p < 0.01.

Evaluation of the impact of BJP-2 on the cell viability of HepG2 cells stimulated for 48 h by MTT assay. As seen in Figure 3A, BJP-2 exhibited significant toxicity at concentrations of 400 and 800 μg/ml, with cell viability of 81.2 ± 2.1% and 55.0 ± 4.0%, respectively (*p* < 0.01). Results showed that BJP-2 inhibited the growth of HepG2 cells within a certain range, and the effect was gradually enhanced with increasing concentration. All together, three optimal concentrations of BJP-2 (200, 400, 800 μg/ml) were selected for subsequent experiments. BJP-2 inhibited tumor cell growth in a similar trend to other polysaccharides. For example, the *Polygonum multiflorum* polysaccharides at 400 μg/ml inhibited the growth of HepG-2 cells by 53.35%; and the mango pomace polysaccharide was active against HepG-2 cells in a quantitatively dependent manner (35, 36).

#### The effect of BJP-2 on apoptosis

Triggering tumor decline by enhancing apoptosis of cancer cells is one of the roles of antitumor drugs (37). Therefore, we detected the apoptosis of HepG-2 cells at different concentrations of BJP-2 using flow cytometry. As presented in Figure 3B, in comparison with the blank control group (5.11 ± 0.86%), the apoptosis rates increased to 20.81 ± 1.3% and 45.72 ± 3.8% by 400 and 800 μg/ml BJP-2 action on HepG-2 cells for 48 h (*p* < 0.01). No significant effect of low doses of BJP-2 (200 μg/ml) was observed, which is consistent with the results of cell viability. After stimulation of HepG2 cells with BJP-2 of 800 μg/ml, the early (Annexin V-FITC+/PI–) and late (Annexin V-FITC+/PI+) apoptosis rates were 25.05 and 25.01 %, respectively, which were significantly higher than that of the control group at 2.24 and 2.90% (Figure 3C). In summary, BJP-2 (800 μg/ml) could cause more than 40% of HepG-2 cells to enter an apoptotic state, suggesting that the induced apoptosis might be one of the mechanisms of action.

#### BJP-2 suppressed HepG-2 cell migration

**Figure 4:**
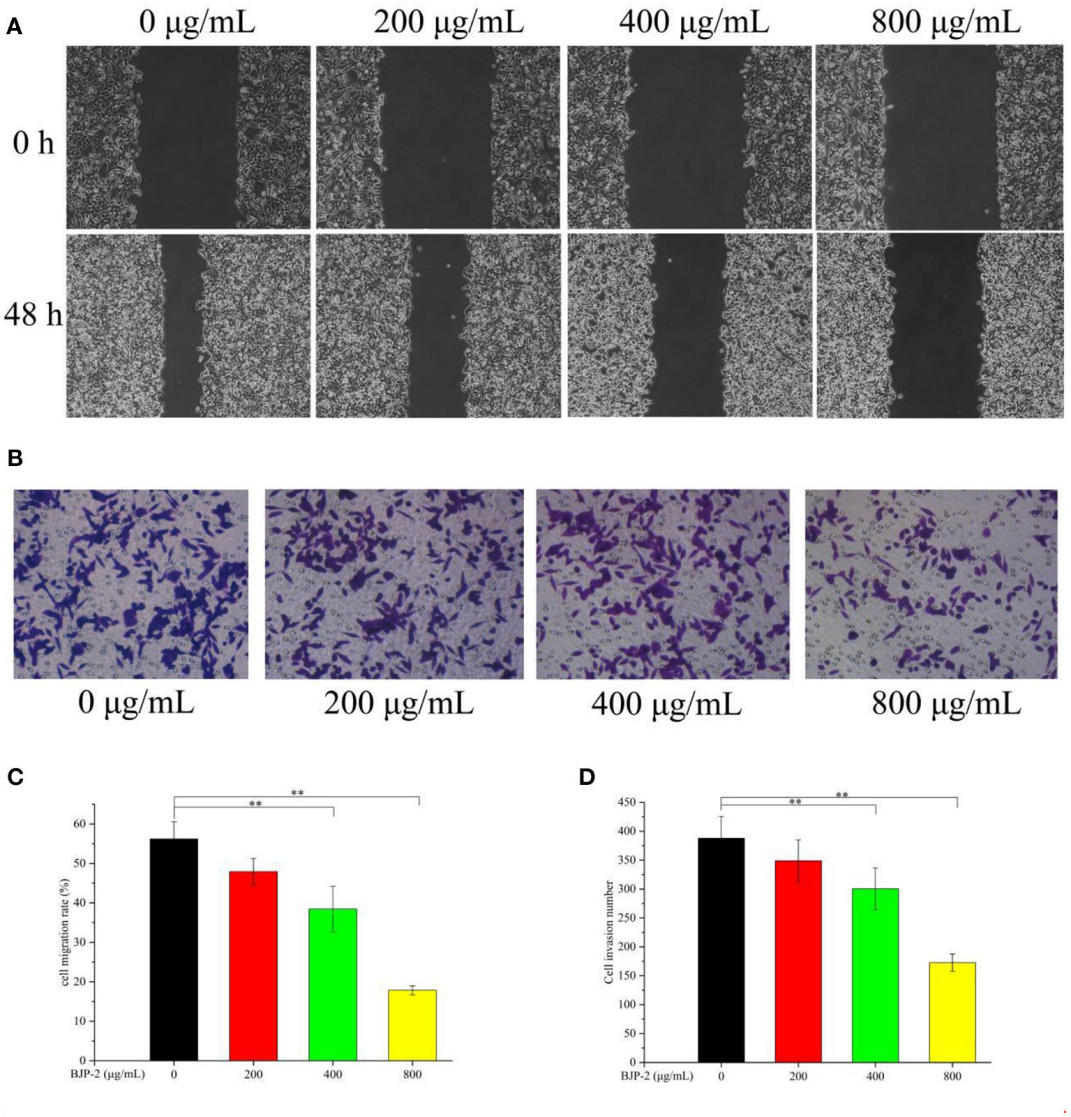
**(A)** The changes of BJP-2 on the migration of HepG2 cells were observed by a inverted phase contrast microscope (100 ×). **(B)** The effects of invasion on HepG2 cells after BJP-2 stimulation were observed by a inverted phase contrast microscope (200 ×). **(C)** Column bar graph of migration rate of HepG2 cells. **(D)** Bar graph summarizes the number of invasion.

One of the key issues in treating cancer is to prevent tumor cells from invading and metastasizing (38). The scratch assay and Transwell assay were applied to detect the effect of BJP-2 on the migration of HepG-2 cells. As shown in Figures 4A,B, supplementation of BJP-2 inhibited the ability of HepG2 cells to heal wounds and cross over to the lower chamber, in contrast to the blank control. Figure 4C showed that the cell migration rate in the control group was 56.20 ± 4.4%, which were significantly decreased to 38.45 ± 5.7% (*p* < 0.01) and 17.86 ± 2.1% (*p* < 0.01) due to the treatment with high doses of BJP-2 (400 and 800 μg/ml). The numbers of cell invasion were 300 ± 35 and 172 ± 14 for BJP-2 at 400 and 800 μg/ml concentrations, respectively, which was significantly lower than that of 387 ± 38 for the control group (*p* < 0.01) (Figure 4D). It was demonstrated that BJP-2 possessed the ability to block the migration and invasion of HepG-2 cells, which inferred that the mechanism might be related to the inhibition of epithelial-mesenchymal transition (EMT). EMT in cancers allows cells to detach from the original tumor tissue and may cause an invasion-metastasis cascade (39). Various polysaccharides have demonstrated anti-tumor mechanisms by inhibiting EMT, such as Ganoderma lucidum polysaccharides, Huaier polysaccharides and Se-lentinan (40–42).

#### BJP-2 modulated gene expression

**Figure 5:**
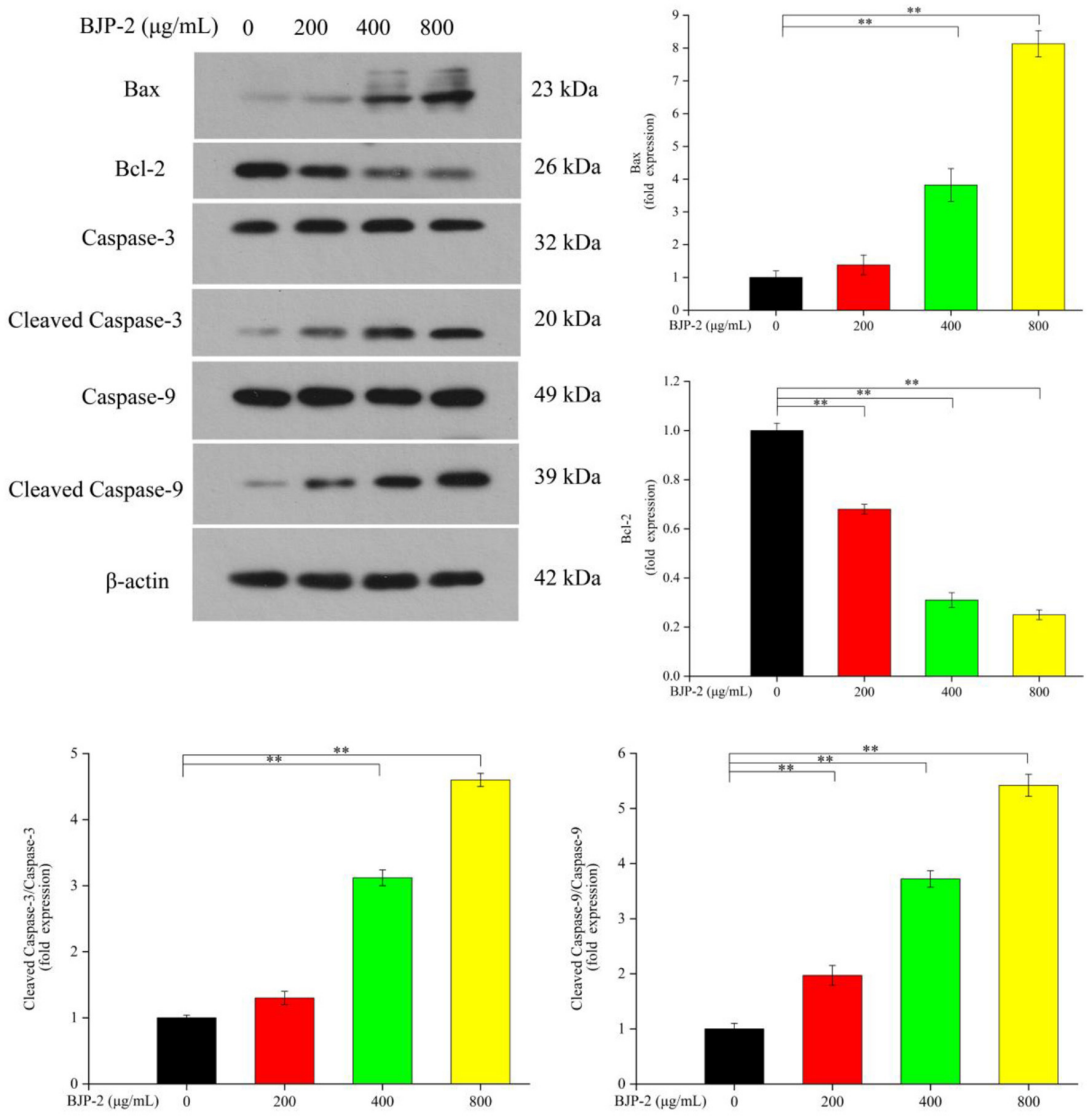
The protein levels of Bax, Bcl-2, Caspase-3, Cleaved Caspase-3, Caspase-9, Cleaved Caspase-9, β-actin by different concentrations of BJP-2 for 48 h. Values are expressed as mean ± SD. *p < 0.05; **p < 0.01.

Promoting apoptosis, a key mechanism by which plant polysaccharides exert their antitumor effects, its critical pathways includes the mitochondrial pathway (43). As known, the pro-apoptotic factor Bax and the anti-apoptotic factor Bcl-2 are antagonistic to each other, where Bax promotes tumor cell apoptosis by promoting the release of cytochrome C, while Bcl-2 does the opposite. Furthermore, the activated Caspase-9 convenes and activates Caspase-3, which is a major executor of apoptosis, thereby triggering a Caspase cascade response to induce apoptosis in tumor cells (44). By Western blot method, the changes of apoptosis-related protein expression in HepG-2 cells after stimulation with different concentrations of BJP-2 were investigated. From Figure 5, as compared with the control group, there were dose-dependent increases in Bax, Cleaved Caspase-3/Caspase-3, Cleaved Caspase-9/Caspase-9 levels on HepG-2 cells after BJP-2 treatment, especially in the high dose group (800 μg/ml) by 2.54-fold, 7.6-fold and 33.52-fold, respectively (*p* < 0.01). By contrast, the expression of Bcl-2 was significantly reduced (*p* < 0.01). Other polysaccharides with similar mode of antitumor effects were also found, such as those derived from Grifola frondosa, *Boletus edulis* and Ganoderma applanatum (45–47).

## Conclusion

In summary, BJP-2 is an acidic polysaccharide with a molecular weight of 6.42 × 10^4^ Da, mainly composed of GalA, Ara, Gal and Rha, which was extracted and isolated from blackened jujube. And its main chain consisted of → 5)-α-L-Ara*f* (1 → 4)-β-D-Gal(1 → , T-α-L-Ara*f* (1 → 4)-β-D-Gal(1 → , and → 4)-α-L-6MeGalA*p*(1 → . Furthermore, the present study demonstrated for the first time that blackened jujube polysaccharides could inhibit the proliferation of tumor cells causing apoptosis through the mitochondrial pathway with a certain degree of dose-dependence. The western blotting analysis indicated that BJP-2 could regulate the expression of apoptosis-related proteins such as Bax, Cleaved Caspase-3, Caspase-3, Cleaved Caspase-9, Caspase-9 and Bcl-2. In addition, BJP-2 initially expressed the ability to suppress the migration and invasion of HepG-2 cells, of which further validation by subsequent experiments was required. It is well know that the biological activities of polysaccharides are closely related to their molecular weight, monosaccharide composition, glycosidic bond type and other chemical properties. On the one hand, high molecular weight plays an important role in enhancing the immune and antitumor activity of polysaccharides. On the other hand, the higher the Glc content, the stronger the anttumor activity. Besides, studies have shown that the activity of the α configuration was poor, while the activity of the β configuration polysaccharide was good. All of these results demonstrated the anti-tumor activity of BJP-2 and presented a theoretical support for further study on the structure-activity relationship. Therefore, there is potential value for BJP-2 to be developed as a new antitumor agent drug against hepatocellular carcinoma. Meanwhile, other anti-tumor mechanisms of blackened jujube polysaccharides are still to be studied and revealed in depth and systematically.

## Data availability statement

The raw data supporting the conclusions of this article will be made available by the authors, without undue reservation.

## Author contributions

GZ and CL: conceptualization, data curation, writing—original draft, software, resources, and investigation. RZ: writing—reviewing and editing and supervision. All authors contributed to the article and approved the submitted version.

## Funding

This work was supported by the Central Guidance on Local Science and Technology Development Program (YDZX2021071), Shandong Province Key Research and Development Program (Rural revitalization of science and technology innovation boosting action) (2021TZXD011); Dezhou Health Food Industry Innovation and Entrepreneurship Community, and Shandong Land and Rural Revitalization Group Co., Ltd., Project Key Technology Integration and Industrialization Demonstration of the Whole Industry Chain for Efficient Green Production and High Value Utilization of Jujube.

## Conflict of interest

The authors declare that the research was conducted in the absence of any commercial or financial relationships that could be construed as a potential conflict of interest.

## Publisher's note

All claims expressed in this article are solely those of the authors and do not necessarily represent those of their affiliated organizations, or those of the publisher, the editors and the reviewers. Any product that may be evaluated in this article, or claim that may be made by its manufacturer, is not guaranteed or endorsed by the publisher.
